# Bio-Inspired Compliant Joints and Economic MPC Co-Design for Energy-Efficient, High-Speed Locomotion in Snake-like Robots

**DOI:** 10.3390/biomimetics10060389

**Published:** 2025-06-11

**Authors:** Shuai Zhou, Gengbiao Chen, Mingyu Gong, Jing Liu, Peng Xu, Binshuo Liu, Nian Yin

**Affiliations:** 1 College of Mechanical and Vehicle Engineering, Changsha University of Science and Technology, Changsha 410114, China; zhoushuai@stu.csust.edu.cn (S.Z.); 202303130418@stu.csust.edu.cn (P.X.); yinnianpoe@163.com (N.Y.); 2 International College of Engineering, Changsha University of Science and Technology, Changsha 410114, China; gongmingyu@stu.csust.edu.cn (M.G.); 202427050108@stu.csust.edu.cn (J.L.); liubinshuo@stu.csust.edu.cn (B.L.)

**Keywords:** snake-like robot, compliant joint design, hierarchical neural oscillator network, energy-optimized control framework

## Abstract

Snake-like robots face critical challenges in energy-efficient locomotion and smooth gait transitions, limiting their real-world deployment. This study introduces a bio-inspired compliant joint design integrated with a hierarchical neural oscillator network and an energy-optimized control framework. The joint mimics biological skeletal flexibility using specialized wheeled mechanisms and adaptive parallel linkages, while the control network enables adaptive gait generation and seamless transitions through a phase-smoothing algorithm. Critically, this work adopts a synergistic design philosophy where mechanical components and control parameters are co-optimized through shared dynamic modeling. The proposed predictive control strategy optimizes locomotion speed while minimizing energy consumption. Experimental simulations demonstrate that the method achieves an 18% higher average forward speed (0.0563 m/s vs. 0.0478 m/s) with 7% lower energy use (0.1952 J vs. 0.2107 J) compared to conventional approaches. Physical prototype testing confirms these improvements under real-world conditions, showing a 12.9% speed increase (0.0531 m/s vs. 0.0470 m/s) and 7.3% energy reduction (0.2147 J vs. 0.2317 J). By unifying mechanical flexibility and adaptive control parameter tuning, this work bridges dynamic performance and energy efficiency, offering a robust solution for unstructured environments.

## 1. Introduction

Snake-like robots have attracted significant attention due to their limbless, elongated body structure and exceptional flexibility, which equips them with the capacity to navigate complex terrains, move through narrow spaces, and surmount obstacles [1]. These capabilities make them highly suitable for applications such as search and rescue, inspection, and environmental monitoring [2,3,4]. However, despite their potential, the practical deployment of snake-like robots faces two critical challenges: energy-efficient locomotion and smooth gait transitions [5]. Addressing these issues is crucial for improving their operational efficiency and adaptability in real-world scenarios.

### 1.1. Challenges in Snake-like Robot Design and Control

The primary challenge lies in balancing energy consumption and locomotion speed, particularly in environments with limited continuous power supply. Traditional control methods often focus on optimizing either energy efficiency [6] or speed, neglecting the trade-off between the two. This shortcoming severely curtails the robot’s operational lifespan and functionality in practical settings. Additionally, snake-like robots have limited carrying capacity, and lower locomotion efficiency. Furthermore, they have restricted sensor integration, which makes it difficult to implement adaptive control and sense complex terrains [7,8].

Another critical issue is the lack of smooth gait transitions. Although current snake-like robots are capable of executing various locomotion gaits, they often struggle with seamless transitions between these gaits. Snake-like robots are composed of multiple modular joints connected in series, forming a highly redundant dynamic system. The movement of adjacent joints is strongly coupled through rigid connecting rods, resulting in nonlinear sudden changes in the inertial force and binding force between the joints during gait switching, which is prone to cause mechanical vibration or even instability. The traditional single-chain CPG network generates gait signals through Hopf oscillators but lacks the smooth processing of parameter transitions. Directly switching the phase difference parameters will lead to discontinuous control signals. Moreover, their ability to select the most appropriate gaits for diverse environments remains underdeveloped, further limiting their adaptability.

### 1.2. Advances in Joint Design and Gait Control

Inspired by the skeletal flexibility of biological snakes, researchers have explored various joint designs to enhance the adaptability of snake-like robots [9,10]. Early developments, such as Hirose’s ACM-III robot, featured single-degree-of-freedom (DOF) joints for basic locomotion [11]. Subsequent innovations, such as the ACM-R4 and ACM-R5 robots, incorporated passive and active wheels to improve mobility [12]. More recently, Shammas et al. designed a three-DOF joint for hyper-redundant robots, enabling 120° motion on a spherical surface and significantly improving environmental adaptability [13]. These advancements emphasize the importance of mechanical design in enhancing robot performance.

Gait control is another key area of research. Traditional methods, such as Serpenoid curve-based control, generate joint reference angles by adjusting parameters like amplitude, frequency, and phase difference [14]. While effective, this approach requires real-time parameter estimation and optimization, increasing computational burden and limiting adaptability to dynamic environments [15]. In recent years, significant progress has been made in the research of bionic technology based on complex system theory. Arena and other scholars successfully simulated the central pattern generator (CPG) of insect movement through a cellular nonlinear network (CNN) architecture, verifying the feasibility of generating global rhythms through self-organization using local connection units [16].

Central pattern generator (CPG)-based control, inspired by biological neural networks, offers a more robust solution [17]. For instance, Liu et al. proposed a unified CPG model using Hopf oscillators to achieve diverse locomotion gaits [18], while Barron-Zambrano et al. developed configurable CPG networks for hexapod robots, albeit with resource constraints [19,20]. Despite these advancements, challenges such as reliance on feedback modules and limited adaptability remain [21].

### 1.3. Optimization of Energy Efficiency and Performance

Efforts to optimize locomotion in snake-like robots have traditionally focused on either energy consumption or speed, rarely addressing both simultaneously. For example, Yamano et al. explored efficient rolling motion via center-of-gravity shifts but encountered energy consumption issues [22]. Recent studies have introduced advanced optimization algorithms to bridge this gap. Liu et al. combined energy criteria with stability constraints for bipedal robots [23], while Bauer et al. integrated linear torsion springs to reduce energy consumption [24]. Other approaches, such as evolutionary algorithms [25] and improved quantum-inspired optimization [26], have also demonstrated success in balancing energy efficiency with performance. Notably, Rebolledo et al. proposed a co-evolutionary framework to optimize both robot morphology and controller parameters, achieving a balance between speed and energy consumption [27]. Similarly, Chen et al. constructed a low-cost underactuated adaptive robotic hand (UARH) with a linkage–spring telescopic rod–slide mechanism, achieving excellent adaptability and cost-effectiveness in grasping tasks [28]. Fabris et al. proposed a three-degree-of-freedom parallel robot optimization strategy. Taking task placement, execution time, and the lower-arm length into account, their strategy minimizes energy use in high-speed picking and placing operations. This achieves a favorable balance between robot productivity and energy efficiency [29].

### 1.4. Contribution of This Study

This study addresses the aforementioned challenges through three key contributions:

Compliant Joint Design: A bio-inspired actively driven compliant joint structure, combining Mecanum wheels and parallel mechanisms with three motor drive units, is proposed to mimic biological flexibility. The joint’s three degrees of freedom (pitching, steering, telescoping) are actively controlled by motors, enabling precise motion generation while maintaining skeletal-like compliance.

Dual-Chain CPG Network: Enhanced with a linear smoothing algorithm, this network generates adaptive gait patterns and ensures smooth transitions between locomotion modes.

Economic MPC Algorithm: This algorithm optimizes locomotion speed while minimizing energy consumption, bridging the gap between dynamic performance and energy efficiency.

Additionally, the compliant joint design (Section 2) and the control algorithms (Section 3 and Section 4) are not independently developed but jointly optimized through their shared dynamic model. Specifically, mechanical parameters (e.g., joint linkage lengths l_1_,l_2_,l_3_, mass *m*, and friction coefficients *c_n_*, *c_t_*) are calibrated to match the CPG-generated gait patterns (e.g., lateral undulation frequency *ω*), phase difference (*ϕ*), and MPC optimization objectives (speed–energy trade-off). Control parameters (e.g., CPG amplitude *α_h_*, MPC prediction horizon *N_p_*, and weight factor *ε* are tuned based on the joint’s physical constraints. This co-design process ensures that structural compliance enhances control efficiency, while adaptive control algorithms fully exploit the mechanical design’s mobility potential.

Simulations and prototype experiments demonstrate the efficacy of the proposed approach. Experimental simulations demonstrate that the method achieves an 18% higher average forward speed (0.0563 m/s vs. 0.0478 m/s) with 7% lower energy use (0.1952 J vs. 0.2107 J) compared to conventional approaches. Physical prototype testing confirms these improvements under real-world conditions, showing a 12.9% speed increase (0.0531 m/s vs. 0.0470 m/s) and 7.3% energy reduction (0.2147 J vs. 0.2317 J). These results validate the robustness of the solution for real-world applications, such as rescue and exploration tasks.

## 2. Compliant Joint Design

**Figure 1:**
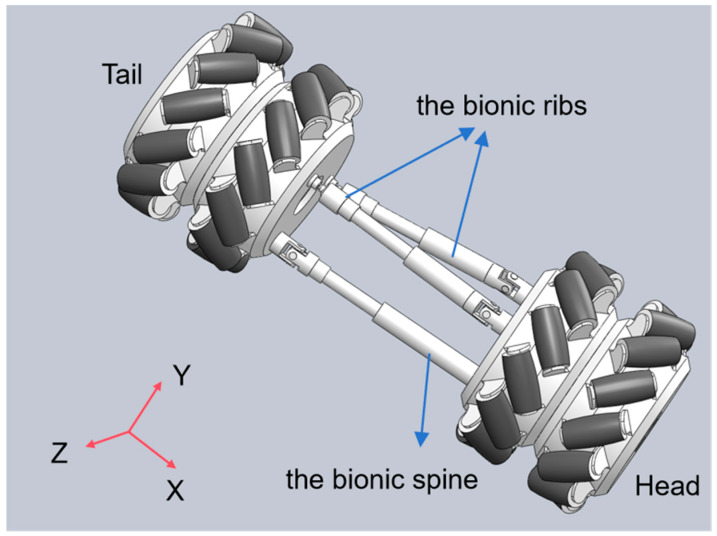
Three-degree-of-freedom compliant joint model.

To enhance the adaptability of snake-like robots to complex environments and enable multimodal motion similar to biological snakes, this paper proposes a three-degree-of-freedom (DOF) compliant joint design. This design aims to mimic the skeletal flexibility of biological snakes, as shown in Figure 1. When the length of the constraint chain and the bionic spine remains unchanged, the pitching motion of the snake-like robot can be achieved by controlling the two bionic ribs chains to extend and retract the same amount. Conversely, steering is realized by controlling the two bionic ribs chains to extend and retract different amounts. When all three chains undergo simultaneous extension and contraction, the overall expansion and contraction motion of the joints is realized. As shown in Figure 1, extension and contraction achieve movement in the X-axis direction, steering achieves rotation along the Y-axis, and pitch achieves rotation along the Z-axis direction, achieving a total of three degrees of freedom.

The joint structure integrates Mecanum wheels with parallel mechanisms, providing the necessary flexibility for diverse locomotion modes. The Mecanum wheel adopts a radial roller structure, supporting compound movement in both longitudinal and transverse directions. It can achieve gait such as lateral fluctuation without the need for the fuselage to turn, breaking through the limitations of traditional wheel systems. Its structure decouples the degrees of freedom of motion, mimics the flexibility of snake-like ribs, actively controls the rolling direction through the UPS mechanism, and optimizes energy transfer with anisotropic friction characteristics, thereby enhancing the propulsion efficiency. These are precisely the capabilities that conventional rigid wheels, omniwheels, or passive rollers are inherently incapable of achieving. Kinematic and dynamic models were developed to study the forces involved and to characterize the robot’s motion, ensuring the design can be optimized for efficient locomotion in a variety of terrain types.

**Figure 2:**
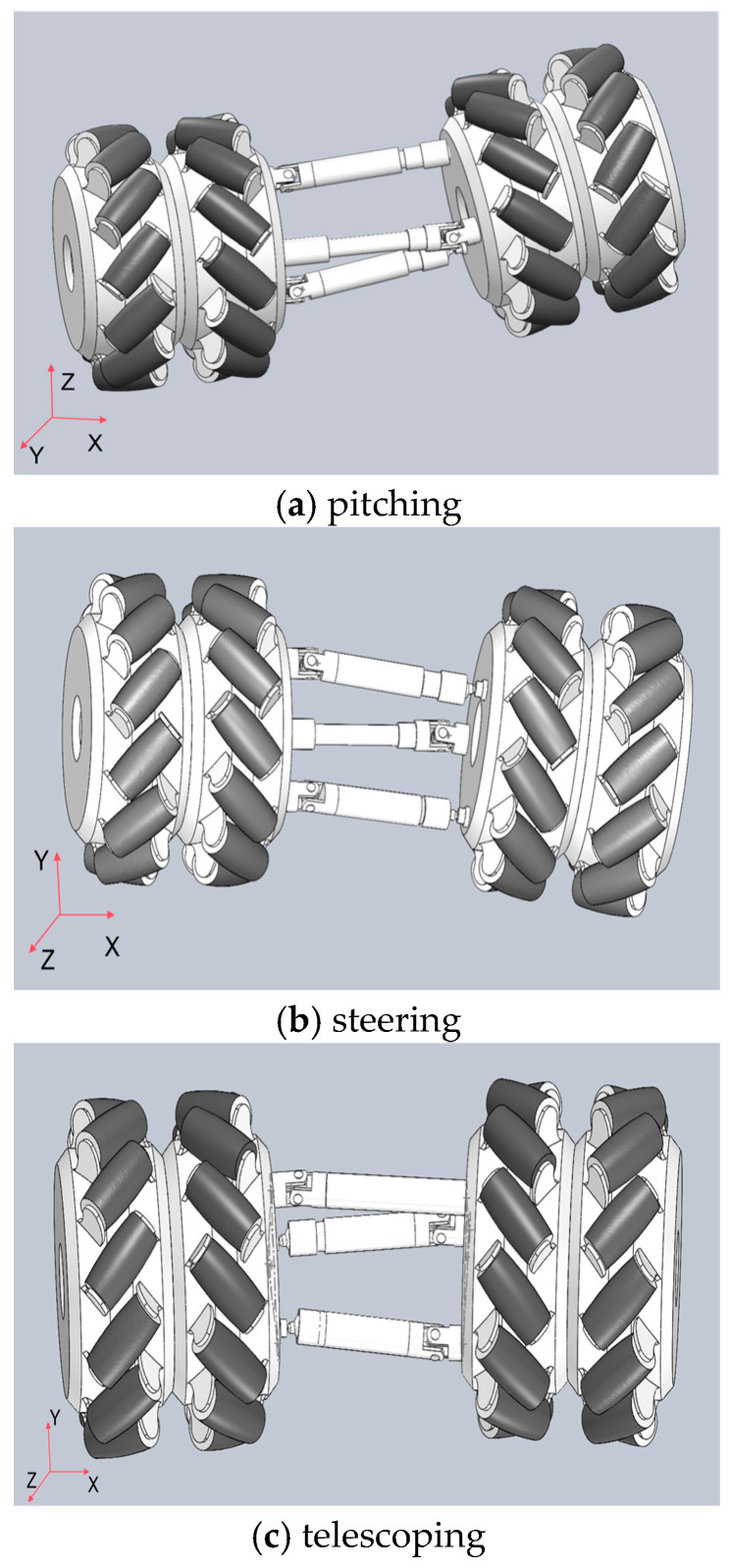
The three motions: pitching, steering, and telescoping.

The snake-like robot features a modular structure with identical joints. As shown in Figure 2, three motor drive units are arranged along three linear struts, enabling the joints to perform three distinct motions: pitching, steering, and telescoping. This decentralized design not only strengthens the overall structure but also enhances load capacity.

**Figure 3:**
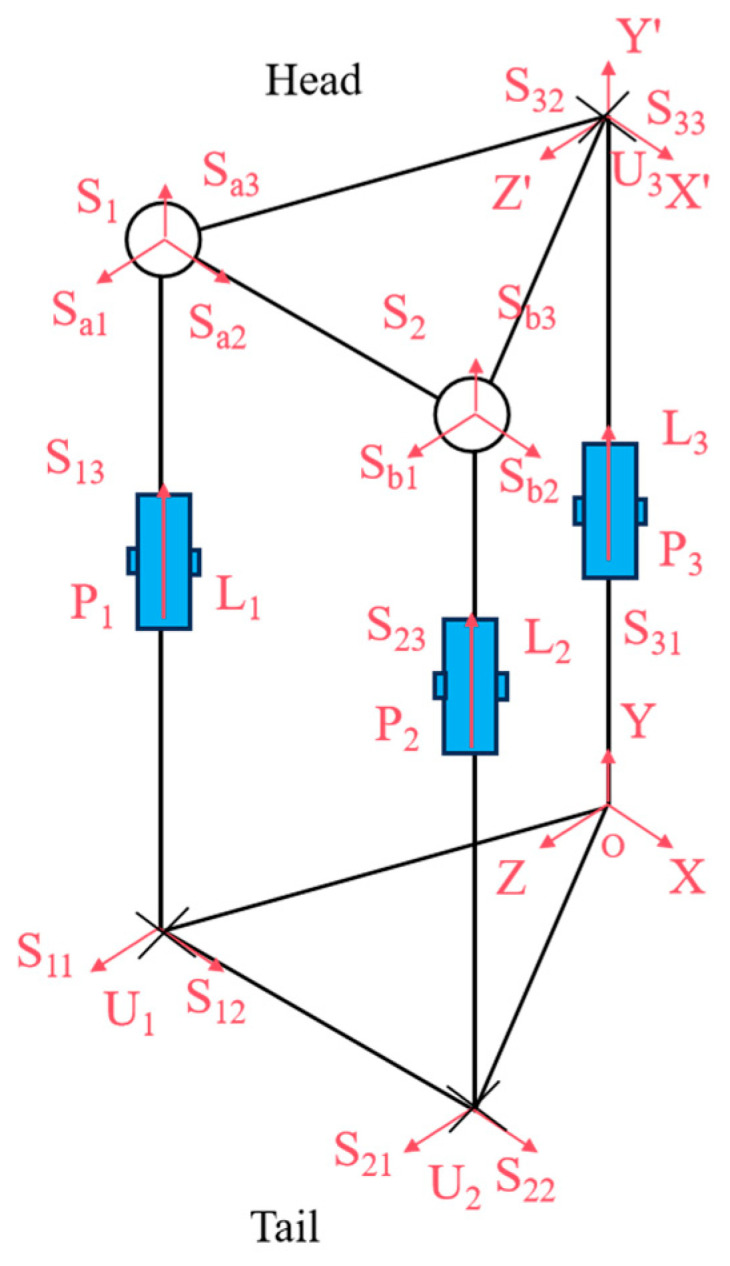
Sketch of compliant joint structure.

As illustrated in Figure 3, we analyzed the kinematic mechanism of the designed flexible joint. This mechanism mimics the rib array distribution and musculoskeletal dynamics found in snakes, achieved through the synergistic configuration of the mobile platform and the prismatic joint. The joint structure consists of two symmetric Hooke’s hinge–cylindrical pair–ball joint mechanisms, which simulate the ribs. Here, U represents Hooke’s hinge, S denotes the ball joint, and P stands for the cylindrical pair. Two symmetric prismatic legs are attached to the ball joint, which effectively counteract the torque perpendicular to the kinematic platform. Additionally, a third strut simulates the bionic spine, providing essential core support. The decoupled control of yaw and pitch motions is facilitated by a specially designed Hooke’s hinge cross-axis system, ensuring smooth motion transfer while maintaining joint length stability.

Within the UPS mechanism, *U_i_* and *S_i_* (I = 1, 2) denote the centers of the Hooke hinge and ball joints, respectively. The constraint center of the constrained-Hooke hinge mechanism is *O*, and the center of the Hooke hinge is *U*_3_. The moving platform is represented by triangle *∆*S_1_*S*_2_*U*_3_, while $\left|\vec{S_{1}U_{3}}\right|=\left|\vec{S_{2}U_{3}}\right|$ defines the stationary platforms, and ∆*U*_1_*U*_2_*O* forms the stationary structure, where $\left|\vec{U_{1}O}\right|=\left|\vec{U_{2}O}\right|$.

A global coordinate system *O*-*XYZ* is established with *O* as the origin, where $X⁄⁄U_{1}U_{2}$, $Z⊥U_{1}U_{2}$, and $Y⊥\Delta U_{1}U_{2}O$. A moving coordinate system is then defined on the moving platform, *U*_3_-*X*’*Y*’*Z*’, where *X*’*Z*’ refers to the direction of the two axes along the Hooke hinge, respectively.

According to spiral theory, any spatial motion can be decomposed into a rigid body rotation around a specific axis and a translation along another axis. The spinor system of motion for joint chain *U*_1_*P*_1_*S*_1_ can then be expressed in global coordinate system *O*-*XYZ* as

(1) $$S_{l1}=\left\{\begin{array}{cccccc} S_{11}=(-1 & 0 & 0 & 0 & 0 & 0{)}^{T}\\ S_{12}=(0 & -\sin\theta_{1} & \cos\theta_{1} & r_{b}\sin\theta_{1} & r_{a}\cos\theta_{1} & r_{a}\sin\theta_{1}{)}^{T}\\ S_{13}=(0 & 0 & 0 & -\sin\varphi_{1} & \cos\varphi_{1}\cos\theta_{1} & \cos\varphi_{1}\sin\theta_{1}{)}^{T}\\ S_{14}=(1 & 0 & 0 & r_{b}+l_{1}\cos\varphi_{1}\sin\theta_{1} & -l_{1}\cos\varphi_{1}\cos\theta_{1} & -l_{1}\sin\varphi_{1}{)}^{T}\\ S_{15}=(0 & 1 & 0 & r_{b}-l_{1}\cos\varphi_{1}\sin\theta_{1} & 0 & -l_{1}\sin\varphi_{1}-r_{a}{)}^{T}\\ S_{16}=(0 & 0 & 1 & l_{1}\cos\varphi_{1}\cos\theta_{1} & l_{1}\sin\varphi_{1}+r_{a} & 0{)}^{T} \end{array}\right.$$

**Figure 4:**
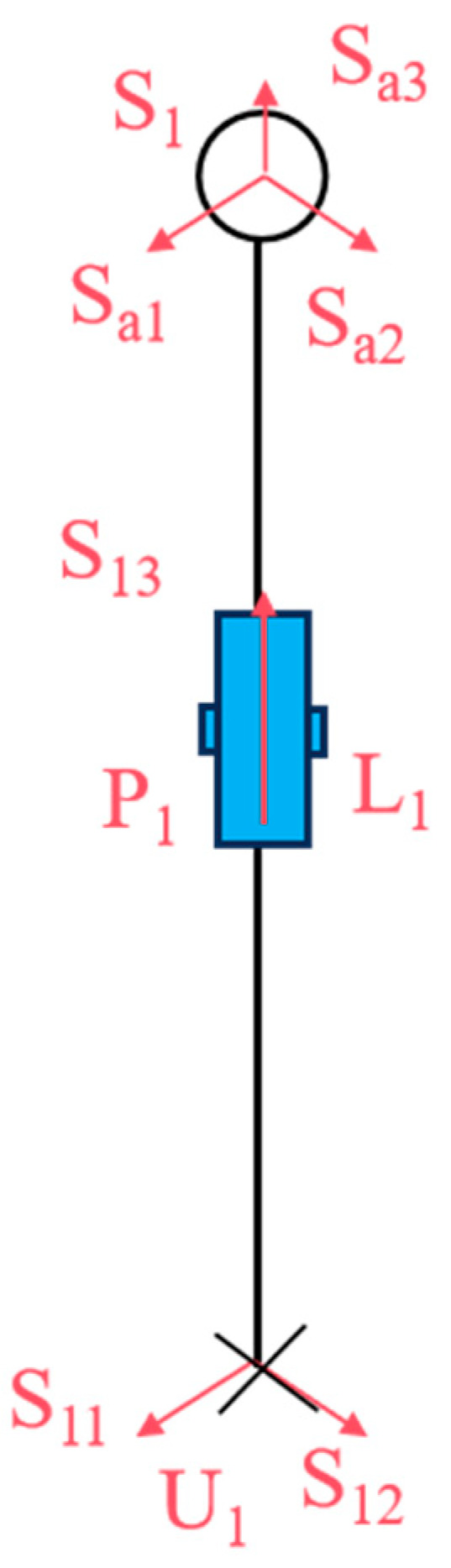
Bionic ribs of U_1_S_1_.

As illustrated in Figure 4, let *S*_11_ and *S*_12_ represent the rotations of the two rotating pairs in the Hooke hinge, *S*_13_ denote the rotations of the cylindrical moving pair, and *S_a_*_1_, *S_a_*_2_, and *S_a_*_3_ represent the rotations of the three rotating pairs in the ball joint. *θ*_1_ and *φ*_1_ denote the rotation angles of the two rotating pairs of the Hooke hinge, respectively, while *l*_1_ represents the length of *U*_1_*S*_1_.

Since the first spin of *S_l_*_1_ is the empty set (i.e., it does not constrain the moving platform), another articulated chain *U*_2_*P*_2_*S*_2_ undergoes the same kinematic analysis as the first chain.

In the global coordinate system *O*-*XYZ*, the position of *U*_3_ is denoted as (0, *l*_3_, 0), where *l*_3_ is the length of *OU*_3_. The rotational quantity of the motion of the Hooke hinge in the *OU*_3_ chain can be expressed as

(2) $$S_{l3}=\left\{\begin{array}{l} S_{31}={\left(\begin{array}{cccccc} 1 & 0 & 0 & 0 & 0 & l_{3} \end{array}\right)}^{T}\\ S_{32}={\left(\begin{array}{cccccc} 0 & -\sin\beta & \cos\beta & l_{3}\cos\beta & 0 & 0 \end{array}\right)}^{T} \end{array}\right.$$

where *β* is the angle of rotation around the *X*’ axis in Hooke hinge joint *U*_3_.

**Figure 5:**
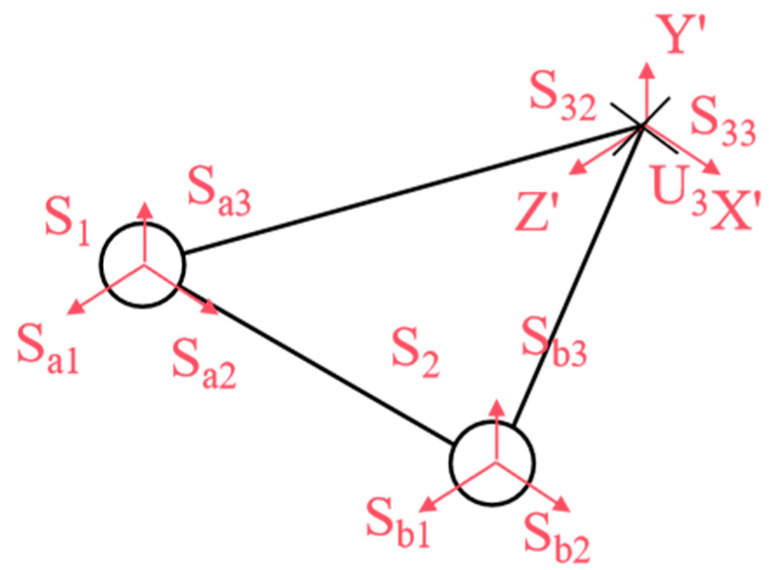
Moving platform ∆*S*_1_*S*_2_*U*_3_.

Since the two UPS chains do not impose constraints on the moving platform ∆*S*_1_*S*_2_*U*_3_, as shown in Figure 5, the motion of the moving platform follows the same rotational behavior as described by Equation (2). Consequently, the moving platform boasts two rotational degrees of freedom, with its center of rotation coinciding with the Hooke hinge *U_3_* center.

The reciprocal rotation of the moving platform relative to *S*_13_ and the fixed platform can be expressed as

(3) $$S^{r}=\left\{\begin{array}{l} S_{1}^{r}={\left(\begin{array}{cccccc} 1 & 0 & 0 & 0 & 0 & -l_{3} \end{array}\right)}^{T}\\ S_{2}^{r}={\left(\begin{array}{cccccc} 0 & 1 & 0 & 0 & 0 & 0 \end{array}\right)}^{T}\\ S_{3}^{r}={\left(\begin{array}{cccccc} 0 & 0 & 1 & -l_{3} & 0 & 0 \end{array}\right)}^{T}\\ S_{4}^{r}={\left(\begin{array}{cccccc} 0 & 0 & 0 & 0 & \cos\beta & \sin\beta \end{array}\right)}^{T} \end{array}\right.$$

From Equation (3), it is evident that the $S_{1}^{r},\;S_{2}^{r},\;S_{3}^{r}$ rotation of the *OU*_3_ chain intersects with the motion rotations *S*_31_ and *S*_32_ at point *U*_3_, while $S_{4}^{r}$ is perpendicular to both *S*_31_ and *S*_32_. The *OU*_3_ chain generates constraints in the *X*’, *Y*’, and *Z*’ directions, as well as a rotational constraint around the *Z*’ axis. However, the two UPS chains do not impose any movement constraints on the platform. This indicates that the primary constraints arise from the *OU_3_* chain, which aligns with the movement constraints of the spine in biological snakes.

**Figure 6:**
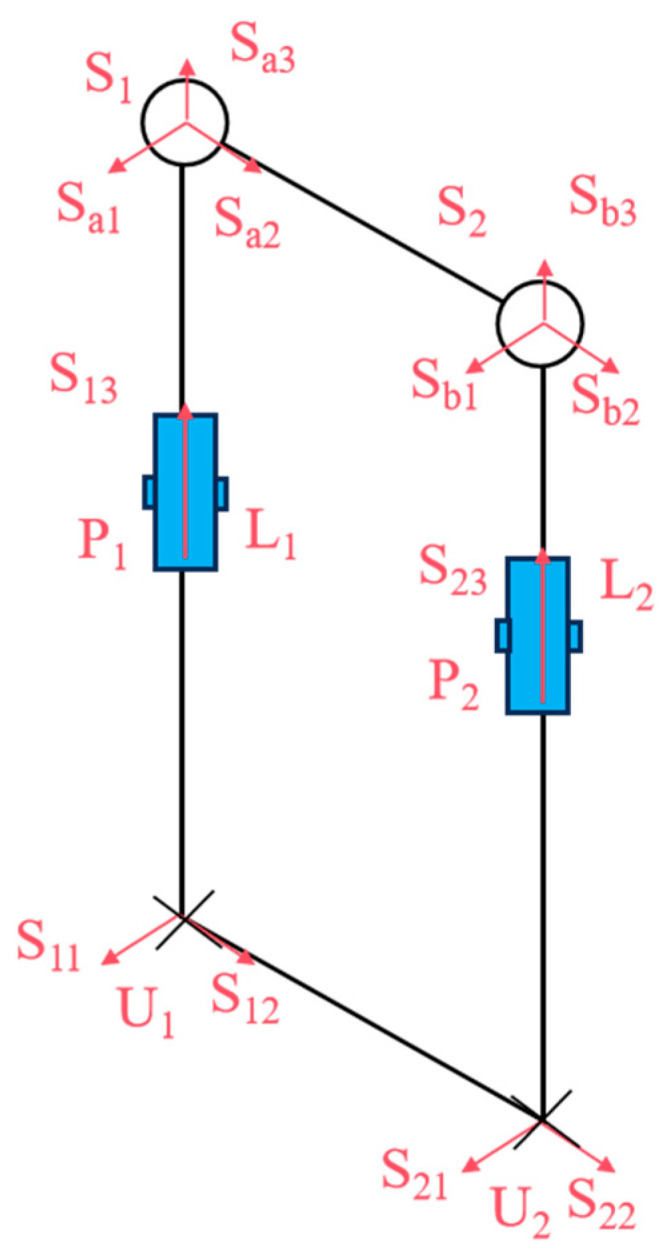
Bionic ribs.

The snake-like compliant joint movable platform exhibits the same two rotational degrees of freedom as the Hooke hinge, specifically the pitching and steering degrees of freedom. In this study, *P*_1_ and *P*_2_ of the UPS chain (the bionic ribs), as shown in Figure 6, were selected as the two driving elements to actuate the motion rotations *S*_13_ and *S*_23_ of the moving platform.

**Figure 7:**
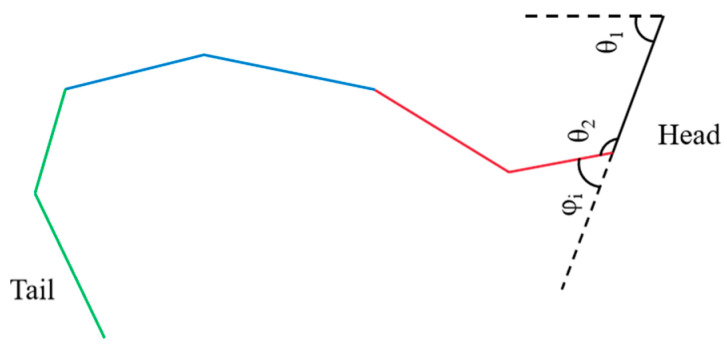
Diagram of the movement trajectory of a biosnake.

Hirose’s study [10] suggests that biological snakes achieve locomotion by twisting their bodies, and their trajectory can be modeled as a trigonometric curve. The direction of movement corresponds to the change in the angular axis of the sine function, as illustrated in Figure 7.

The motion trajectory can be described as

(4) $$\begin{array}{l} x(s)=\int_{0}^{s}\cos(\tau )d\sigma \\ y(s)=\int_{0}^{s}\sin(\tau )d\sigma \\ \tau =a\cos(bs)+c\sigma \end{array}$$

In this case, *a*, *b*, and *c* represent the parameters of the snake’s motion curve, and *s* represents the arc length in the forward direction. Equation (4) presents a continuous curve; however, to ensure effective motion control of the snake-like robot, the curve equation necessitates further refinement.

To attain control of the snake-like robot, the continuous control angles of the rotating joints need to be ascertained:

(5) $$\varphi_{i}=\alpha \cos(\omega t+i\beta )+\gamma$$

Let *φ_i_* represent the control angle of the first segment, and $a\sin\left(\frac{\beta }{n}\right),\beta =\frac{b}{n},\gamma =\frac{c}{n}$. When *b* = 2π, the robot completes one full motion cycle, and the control angle can be expressed as *φ_i_* = *θ_i_*_+1_ − *θ_i_*, where *θ_i_* is the angle between the joint and the X-axis.

Angle *θ*_1_ can be derived when the snake-like robot completes one full gait cycle:

(6) $$\theta_{1}=\frac{1}{2}\left(\frac{k}{n}\pi -\sum_{i=1}^{k/2}\varphi_{i}\right)\;\left\{\begin{array}{ll} k=n & n\;is\;an\;even\;number\\ k=n-1 & n\;is\;an\;odd\;number \end{array}\right.$$

where *K* is the cycle parameter determined by the number of joint modules in the snake-like robot. The amplitude and wavelength of the robot’s motion can then be calculated as

(7) $$\begin{array}{l} A=\frac{1}{2}\sum_{i=1}^{n}|\sin(\theta_{i})|\\ \lambda =2l\sum_{i=1}^{n}\cos(\theta_{i}) \end{array}$$

Here, *l* denotes the half-length of the joint, *A* is the maximum theoretical amplitude of the snake-like robot, and the robot’s actual motion should follow the trajectory of its center of mass.

By analyzing the motion control of individual joints, the linear actuator angle control equations for each supple joint of the snake-like robot can be derived:

(8) $$l_{i^{\prime }}=\sqrt{\frac{a^{2}}{2}+l_{0}^{2}-a\sqrt{\frac{a^{2}}{4}+l_{0}^{2}}\cos\left(\arctan\left(\frac{2l_{0}}{a}\right)+\varphi_{i}\right)}\;l_{2^{\prime }}=\sqrt{\frac{a^{2}}{2}+l_{0}^{2}-a\sqrt{\frac{a^{2}}{4}+l_{0}^{2}}\cos\left(\arctan\left(\frac{2l_{0}}{a}\right)-\varphi_{i}\right)}$$

where $l_{1}^{i}$ and $l_{2}^{i}$ are the lengths of the two rods connected to the *i*-th joint, *l*_0_ is the initial length of the rods, *φ_i_* is the horizontal deflection angle of the *i*-th joint, and *a* is the distance between the two rods inside the joint. The pitch angle of the joint is controlled as

(9) $$l_{1}^{i}=l_{2}^{i}=\sqrt{\frac{3a^{2}}{2}+l_{0}^{2}-\sqrt{3}a\cos\left(\arctan\left(\frac{2\sqrt{3}l_{0}}{3a}\right)-\delta_{i}\right)\sqrt{\frac{a^{2}}{2}+l_{0}^{2}}}$$

where *δ_i_* is the pitch angle of the *i*-th joint.

According to the equations of motion, controlling the extension and retraction lengths of the three rods of the flexible joints allows for the realization of expansion, contraction, steering, and pitching movements of a single joint. Specifically, when the length of the constraint chain *OU*_3_ remains unchanged, the pitching motion of the snake-like robot can be achieved by controlling the two UPS chains to extend and retract the same amount. Conversely, steering is realized by controlling the two UPS chains to extend and retract different amounts. When all three chains extend and contract simultaneously, the joint undergoes overall expansion or contraction.

**Figure 8:**
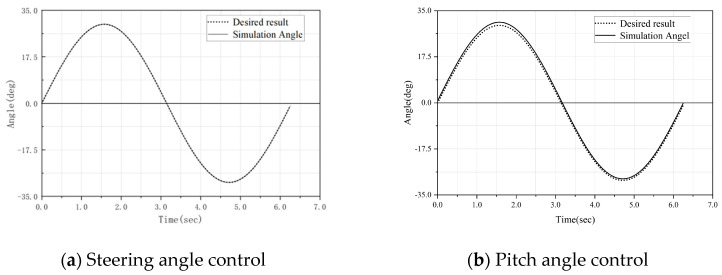
Simulation angle vs. desired angle.

The simulated angles during the motion of the snake-like robot can be obtained through Equations (8) and (9), and were compared with the desired sinusoidal fluctuation motion trajectory. As shown in Figure 8, it can be observed that the steering simulation angles of the robot joints are closely aligned with the desired results and are distributed very uniformly. However, as shown in Figure 8b, with the increase in the pitch angle, the contact distance between the simulated angle and the actual expected angle becomes larger, and the resulting error becomes more obvious.

## 3. Adaptive Gait Control via CPG Network

### 3.1. Dual-Chain CPG Architecture

Central pattern generator (CPG) networks, which utilize specialized neural oscillators to generate gait signals for snake-like robots, offer significant advantages in environmental adaptability through their built-in feedback mechanisms. However, designing these models and adjusting their parameters remain challenging tasks.

To address these challenges, we propose a novel dual-chain CPG architecture based on Hopf neural oscillators. This design takes advantage of the periodic nature of serpentine locomotion to achieve three key benefits:

1. Multimodal Gait Generation: The network generates a variety of locomotion patterns while reducing the dimensionality of control parameters, a limitation in single-chain models where high-dimensional outputs require complex parameter tuning. A small set of state variables can govern high-dimensional joint motions, contrasting with traditional single-chain models that often rely on the independent adjustment of each joint.

2. Enhanced Dynamical Properties: The use of natural rhythmic signals for joint motion control, mimicking biological neural dynamics and avoiding the discontinuous signals of non-oscillatory models’ faster convergence rates to stable gaits, significantly reduces the convergence time compared to single-chain Hopf networks. This simplified parameter transitions via diffusive coupling, whereas a traditional single-chain CPG suffers from abrupt changes in control signals during gait switching.

3. Implementation Efficiency: The dual-chain architecture significantly reduces computational complexity during gait parameter optimization, making it more efficient in real-time computation than single-chain models with equivalent performance. This efficiency addresses the computational bottleneck of traditional high-dimensional CPG networks.

The kinetic equation for the Hopf neural oscillator is given by

(10) $$\begin{array}{c} F_{h}(a,b)=\left[\begin{array}{c} -\gamma \left(\frac{f{\left(a\right)}^{2}+f{\left(b\right)}^{2}}{r_{x}^{2}}-\zeta \right)f(a)+\tau (t)f(b)\\ -\tau (t)f(a)-\gamma \left(\frac{f{\left(a\right)}^{2}+f{\left(b\right)}^{2}}{r_{x}^{2}}-\zeta \right)f(b) \end{array}\right]\\ f(a)=a-a_{c}\\ f(b)=b-b_{c} \end{array}$$

Among them, x = (a,b)^T^, *r_x_* is the radius of the limit circle range, *v* is the convergence speed that affects the output value to converge to the limit circle, *a_c_* and *b_c_* determine the center position within the limit circle range, *ε* is the bifurcation parameter of the neuron, and *ζ =* 1 is set in this paper.

Here, *a_xi_* is the output value of the neural oscillator, and *a_i_* and *b_i_* offsets are determined by *a_c_*_,*i*_ and *b_c_*_,*i*_, respectively. *a_c_*_,*i*_ and *b_c_*_,*i*_ are the core control parameters, which are the offset parameters of the horizontal and vertical centers, respectively, determining the reference position of the oscillator on the horizontal axis (A-axis) and the vertical axis (B-axis). The output value of Hopf’s neural oscillator can be stabilized to converge to the a-b plane, where a and b are sinusoidal curves after the stabilization of the output value.

In order to realize the three-dimensional motion control of the snake-like robot in this study, a two-chain Hopf neural oscillator network was designed based on the Hopf neural oscillator network of reference [30]. The dynamics equation of its first *i* oscillator is expressed as

(11) $${\dot{x}}_{i}=F_{h}(x_{i})-\alpha \sum_{j}\omega_{i,j}\left(x_{i}-\frac{A_{xi}}{A_{xj}}R(\phi_{ij})x_{j}\right)\;{\dot{y}}_{i}=F_{h}(y_{i})-\beta \left(y_{i}-\frac{A_{xi}}{A_{xj}}R(\phi_{yx})x_{i}\right)\;R(\varphi )=\left[\begin{array}{cc} \cos\varphi_{ij} & -\sin\varphi_{ij}\\ \sin\varphi_{ij} & \cos\varphi_{ij} \end{array}\right]$$

where x_i_ = [a_xi_,b_xi_]^T^ and y_i_ = [a_yi_,b_yi_]^T^ are the outputs of the oscillator in the horizontal and vertical directions, respectively, and *a_xi_* and *b_xi_* represent the serpenoid composite curves of the control signals for the *i*-th steering joint and the *i*-th pitch joint, respectively.

**Figure 9:**
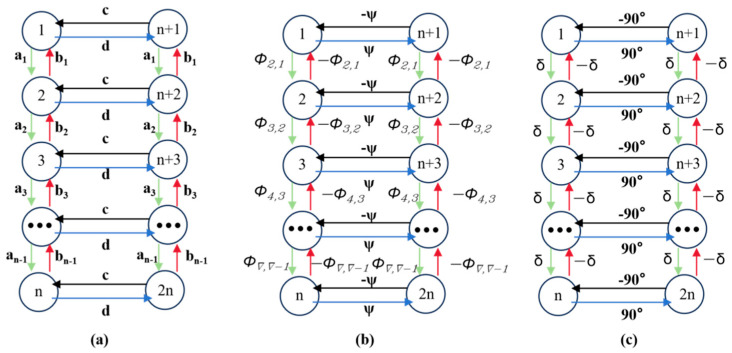
Structure of double-chained Hopf neural oscillatory network. (**a**) Weighting factors between neighboring neurons; (**b**) uniform phase difference among adjacent neurons; (**c**) special CPG architecture with uniform phase difference among neighboring neurons.

The structure of the dual-chain Hopf neural oscillatory network is shown in Figure 9. The left green chain and right red chain of Hopf oscillators generate reference curves for horizontal and vertical joint motions, respectively. Here, *a*, *b*, *c*, and *d* are the weights between neighboring neurons, and *δ* represents the phase difference between adjacent neurons, which varies depending on the specific motion being executed.

**Figure 10:**
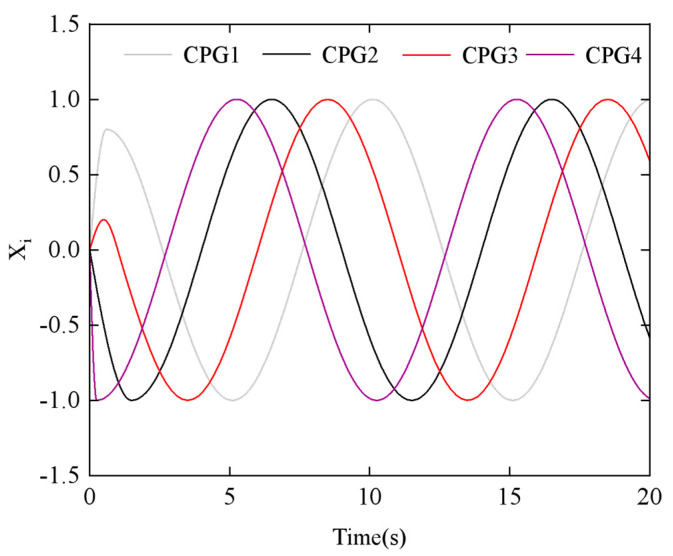
Steering joint control signal generated by the CPG network.

The dual-chain CPG neural oscillatory network is composed of 2*n* Hopf oscillators. The left CPG chain is numbered 1 to *n*, controlling the joint movement in the horizontal direction of the snake body, and the right chain is numbered from *n* + 1 to 2*n*, controlling the joint movement in the vertical direction of the snake body. All oscillators are connected via diffusive coupling. Various gaits of the snake-like robot can be achieved by adjusting the phase difference in the CPG network. In this study, the lateral undulating gait was simulated using the parameters in Table 1. Figure 10 illustrates how the output signals of the four CPG oscillators change when the phase difference *ϕ_h_*_,*ij*_ = π/3.

**Figure 11:**
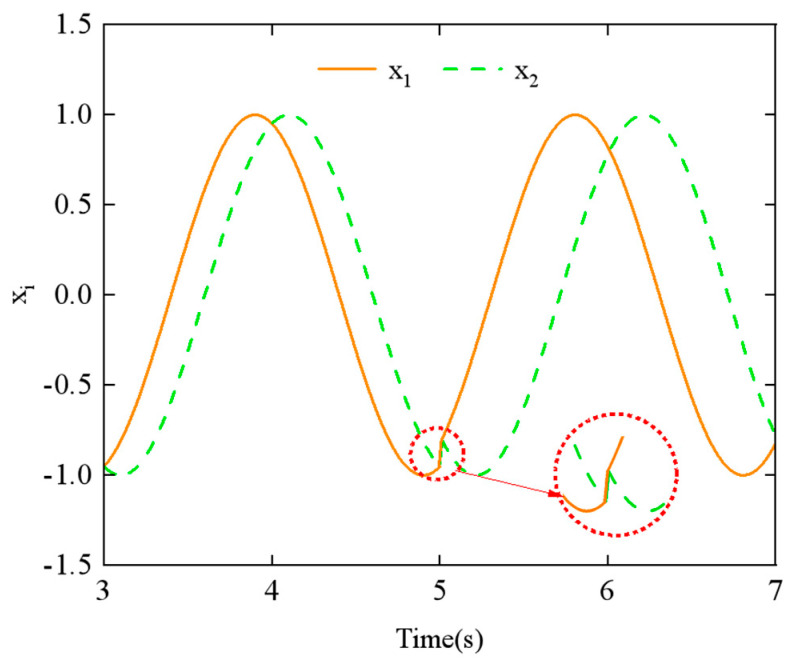
Effect of varying the phase difference *ϕ_h_*_,*ij*_ parameter on the output of the CPG network.

**Figure 12:**
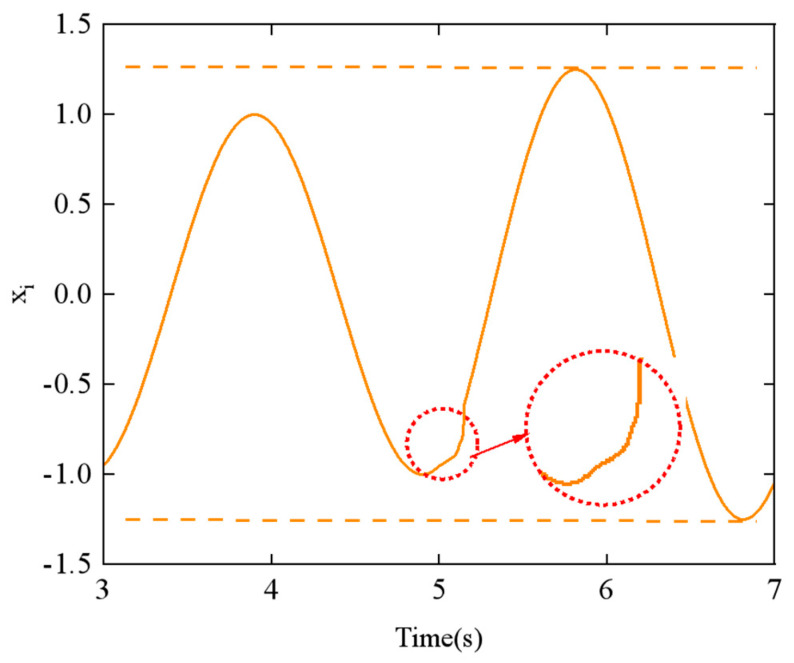
Effect of varying the amplitude *α_h_* parameter on the output of the CPG network.

To explore the relationship between the parameters and the CPG output signals, we examined the effects of the phase difference *ϕ_h_*_,*ij*_ and the amplitude *α_h_* using the step-switching method. Figure 11 shows the simulation results of phase difference *ϕ_h_*_,*ij*_ at *t* = 5 s, with a change from π/4 to π/2. Similarly, Figure 12 displays the simulation results of amplitude *α_h_* at *t* = 5 s, with a change from π/6 to π/3.

From Figure 11 and Figure 12, it can be observed that the CPG output exhibits abrupt and discontinuous curves when the parameters are varied. Such aberrant or discontinuous control signals can lead to uncoordinated motion in the motors of the snake-like robot, potentially causing damage to both the motors and the joint structure. To prevent this issue, we investigated methods to achieve the smooth switching of gait parameters within the CPG network.

### 3.2. Gait Transition Smoothing

Evaluating curve continuity and smoothness mainly depends on parametric continuity (C*_n_*) and geometric continuity (G*_n_*); C_n_ and G_n_ denote the n-th order parametric continuities. Geometric continuity ensures geometric consistency, while parametric continuity demands underlying parameter continuity [31]. In this study, it was essential to guarantee that the smoothness of the snake-like robot’s motion was at least (C_2_) and (G_2_).

To analyze the C_1_ order continuity of the robot’s motion, we simplified the CPG network to a single-phase oscillator model, which is mathematically represented as follows:

(12) $${\dot{\theta }}_{i}=2\pi \nu_{i}+\sum \omega_{ij}\sin(\theta_{j}-\theta_{i})$$

where *θ_i_* is the phase of the *i*-th oscillator, *θ_j_* is the phase of the *j*-th oscillator, *υ_i_* is the intrinsic frequency of the oscillator, and *ω_ij_* represents the coupling weight between neighboring oscillators. According to [32], for smooth output signals in a phase oscillator model, it is crucial that *ω_ij_* exceeds *υ_i_.*

Thus, the oscillation model in Equation (12) is enhanced by introducing a parameter (*τ*), which simultaneously controls both *ω_ij_* and *υ_i_*, as follows:

(13) $$\tau {\dot{\theta }}_{i}=2\pi \nu_{i}+\sum \omega_{ij}\sin\left(\theta_{j}-\theta_{i}-\phi_{ij}\right)$$

where *ϕ_ij_* is the phase difference. To ensure effective control of the CPG output, the value of *τ* should remain less than 1. If *τ* > 1, the output performance is compromised. By adjusting *τ*, smooth output can be achieved while maintaining the stability of *υ_i_* and *ω_ij_*.

The output of a single oscillator is defined as

(14) $$x_{i}=A\cos(\theta_{i})$$

where *x_i_* represents the output signal (i.e., the joint angle), and *A* is the amplitude.

As per the two-chain CPG network in the previous section, all oscillators operate with an identical phase parameter *ϕ* to create symmetric joint motions for the snake-like robot. The total phase difference is set as (−*ϕ*) for downward motion and *ϕ* for upward motion.

(15) $$\phi_{a}=n\phi$$

Representing the total driven joints of the robot, *n* is subject to a total phase difference of 2π. Accordingly, the total joint movements, *N*, of the snake-like robot can be presented as

(16) $$N=\frac{n\phi }{2\pi }$$

By varying the phase difference *ϕ*, different gaits of the snake-like robot can be realized, which is mathematically represented by

(17) $$\phi =\frac{2\pi n}{N}$$

The first-order continuity C_1_ of the CPG network output can be derived from Equation (13) and expressed as

(18) $$\tau {\ddot{\theta }}_{i}=-\omega_{ij}[{\dot{\theta }}_{i}\cos(\theta_{j}-\theta_{i}-\phi_{ij})]$$

Deriving Equation (18) again yields the parametric continuity evaluation C_2_, with the following expression:

(19) $$\tau {\ddot{\theta }}_{i}=\omega_{ij}\left[\left[{{\dot{\theta }}_{i}}^{2}\cos(\theta_{j}-\theta_{i}-\phi_{ij})\right]-{\ddot{\theta }}_{i}\cos(\theta_{j}-\theta_{i}-\phi_{ij})\right]$$

However, for Equations (18) and (19), in assuming that the phase transition occurs from *ϕ_ij =_ ϕ*_1_ to *ϕ_ij =_ ϕ*_2_, neither equation proves to satisfy parametric continuity C_1_ and C_2_. As shown in Figure 11, there is a noticeable discontinuity in the CPG network’s output curve when the phase difference parameter is switched, which also fails to demonstrate the geometric continuity of the switching process.

**Figure 13:**
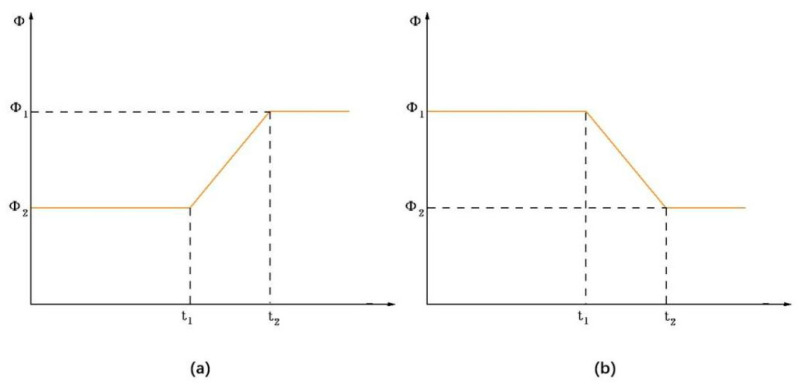
Linear variation in the phase difference *ϕ.* (**a**) Increase in *ϕ*; (**b**) decrease in *ϕ.*

To achieve smooth gait transitions, Qiao et al. [33] designed a double-chain CPG network to generate the control signals for the snake-like robot and proposed a method based on linear segmented functions for gait smoothing. In this paper, we propose an improved linear function algorithm for gait smoothing conversion, introducing a linear bipolar function as the activation function. This modification ensures that the output signal changes linearly during the phase difference switching process. Figure 13 illustrates the variation in phase difference *ϕ* over the time interval *t*_1_ to *t*_2_.

As an example of *ϕ* reduction, the phase difference *ϕ* as a function of time *t* is described by a linear bipolar function:

(20) $$\phi =\left\{\begin{array}{ll} \phi_{1}, & t\leq t_{1}\\ \phi_{1}-a(t_{1}-t), & t_{1}<t<t_{2}\\ \phi_{2}, & t\geq t_{2} \end{array}\right.$$

where $a=\phi_{1}\left(\frac{N_{2}}{N_{1}}\right)\frac{\left(1-\frac{N_{1}}{N_{2}}\right)}{t_{2}-t_{1}}$, *N*_1_ and *N*_2_ represent the number of movements of the joints, *ϕ*_2_ and *N*_2_ are pre-determined values, (*t*_2_ − *t*_1_) is the switching time for the phase difference from *ϕ*_1_ to *ϕ*_2_, and *t*_1_ is the moment when the phase difference begins to change.

With the substitution of Equation (20) into Equations (18) and (19), it is straightforward to show that the output curves exhibit C_1_ and C_2_ continuity of the parameters around (*t* = *t*_1)_ and (*t* = *t*_2_), meaning that the parameter smoothing is improved.

To validate the effect of geometric smoothness, simulation experiments were conducted to evaluate the output signal of the linear smoothing switching algorithm applied to the CPG network. In this experiment, the phase difference of neighboring joints *ϕ_ij_* is used as the adjustment parameter. The initial and final phase differences were set to *ϕ*_1_ *= π/*4 and *ϕ*_2_ *= π/*2, respectively, with (*n* = 8) joints. The switching time, (*t*_2_ − *t*_1_), was set to 1 s.

**Figure 14:**
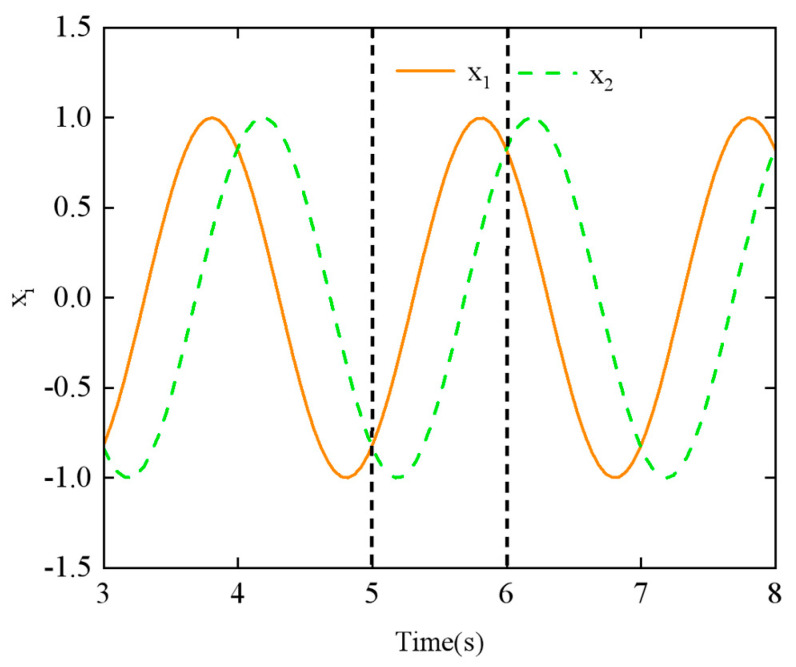
CPG output signal with phase difference adjusted by applying linear smoothing switching algorithm.

The output signal of the CPG network using the linear smoothing switching algorithm is shown in Figure 14. When compared to Figure 11, it is evident that the smoothness of the output signal *x_i_* over time is significantly improved during the transition period between (*t*_1_ = 5 s) and (*t*_2_ = 6 s).

## 4. Economic MPC for Locomotion Optimization

### 4.1. Formulation of Economic MPC

Traditional methods for enhancing snake-like robot locomotion typically prioritize minimizing energy consumption, often at the expense of other performance metrics, such as speed. This limitation hinders their practical applicability. To overcome this challenge, we propose an Economic Model Predictive Control (MPC) framework that optimizes both energy efficiency and locomotion speed simultaneously.

MPC addresses a finite-horizon optimal control problem at each sampling time point, enabling real-time gait parameter optimization. Conventional approaches often involve exhaustive searches over combinations of amplitude and frequency, which can be computationally expensive and lack precision. In contrast, our MPC-based approach efficiently determines the optimal gait parameters, maximizing the robot’s forward speed while minimizing energy consumption, even in complex, dynamic environments.

**Figure 15:**
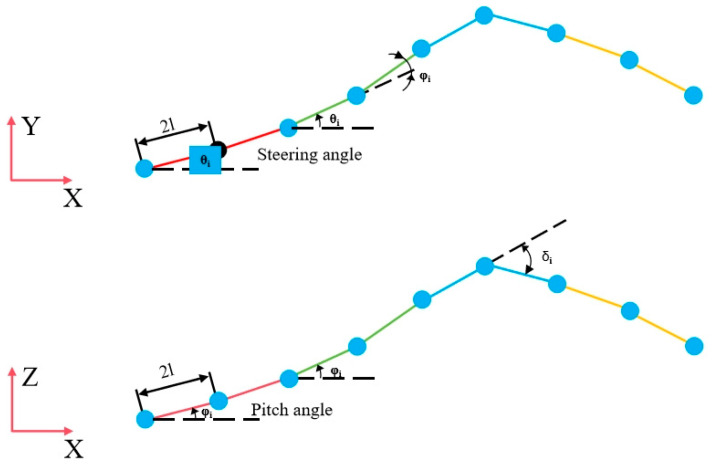
Simplified connecting rod system.

For simplification, the snake-like robot is modeled as having *N_l_* − 1 moving joints linked to *N_l_* rods of uniform mass *m* and length 2*l*. The center of mass of each rod is located at its midpoint. The robot’s motion is driven by *N_l_* − 1 actuators, as shown in Figure 15.

The connection between the joints of the robot must satisfy three complete kinematic constraints, which can be expressed as

(21) $$A=\left[\begin{array}{ccccc} 1 & 1 & & & \\ & \ddots & \ddots & & \\ & & \ddots & \ddots & \\ & & & 1 & 1 \end{array}\right]\in R^{(N_{i}-1)\times N_{i}}\;D=\left[\begin{array}{ccccc} 1 & -1 & & & \\ & \ddots & \ddots & & \\ & & \ddots & \ddots & \\ & & & 1 & -1 \end{array}\right]\in R^{(N_{i}-1)\times N_{i}}\;e=\left[\begin{array}{c} 1\\ \vdots \\ 1 \end{array}\right]\in R^{N_{i}},\;\bar{e}=\left[\begin{array}{c} 1\\ \vdots \\ 1 \end{array}\right]\in R^{N_{i}-1}\;\bar{D}=D^{⊤}{\left(DD^{⊤}\right)}^{-1}\in R^{N_{l}\times (N_{l}-1)}$$

Matrices *A* and *D* correspond to the addition and subtraction of adjacent elements in a vector. The snake-like robot can advance forward via anisotropic viscous ground friction. Specifically, the ground friction perpendicular to the linkage surpasses the ground friction parallel to the linkage.

Therefore, using *c_n_* ϵ *R* > 0 for the normal friction coefficient and *c_t_* ϵ *R* > 0 for the tangential friction coefficient, the push factor is defined as follows.

Matrices *A* and *D* are utilized to represent the addition and subtraction of neighboring elements in a vector.

The snake-like robot’s forward movement is facilitated by anisotropic viscous ground friction, where the perpendicular friction exceeds the parallel friction relative to the linkage. We define the push factor using the following friction coefficients: *c_n_* ϵ *R* > 0 for the normal friction coefficient, and *c_t_* ϵ *R* > 0 for the tangential friction coefficient.

(22) $$c_{p}=\frac{c_{n}-c_{t}}{2l}$$

A prediction model, incorporating both the robot’s state and the surrounding environment, is constructed using the control method proposed in [34]. This model allows for the computation of optimal control parameters at each sampling time *T_s_*. Since the snake-like robot has (*N* + 2) degrees of freedom, the state vector, which includes the robot’s position and velocity, has a dimension of (2*N* + 4). The system state vector can be expressed as

(23) $$x={\left[\begin{array}{c} \phi^{T},\theta ,p_{x},p_{y},v_{\phi }^{T},v_{\theta },v_{t},v_{n} \end{array}\right]}^{T}\in R^{2N+4}$$

Thus, the complete model of the snake-like robot can be expressed as

(24) $$\phi (t+1)=\phi (t)-T_{s}v_{\phi }(t)\;\theta (t+1)=\theta (t)+T_{s}v_{\theta }(t)\;p_{x}(t+1)=p_{x}(t)+T_{s}\left(v_{t}(t)\cos(\theta (t))-v_{n}(t)\sin(\theta (t))\right)\;p_{y}(t+1)=p_{y}(t)+T_{s}\left(v_{t}(t)\sin(\theta (t))+v_{n}(t)\cos(\theta (t))\right)\;v_{\phi }(t+1)=v_{\phi }(t)+T_{s}u(t)$$

$$\begin{array}{ll} v_{\theta }(t+1) & =v_{\theta }(t)+T_{s}\left(-\lambda_{1}v_{\theta }(t)+\frac{\lambda_{2}}{N_{i}-1}v_{t}(t)e^{-T}\phi (t)\right)\;\\ v_{t}(t+1) & =v_{t}(t)+T_{s}\left(-\frac{c_{t}}{m}v_{t}(t)+\frac{2c_{p}}{N_{i}m}v_{n}(t)e^{-T}\phi (t)-\frac{c_{p}}{N_{i}m}\phi^{T}(t)\bar{D}v_{\phi }(t)\right)\;\\ v_{n}(t+1) & =v_{n}(t)+T_{s}\left(-\frac{c_{n}}{m}v_{n}(t)+\frac{2c_{p}}{N_{i}m}v_{t}(t)e^{-T}\phi (t)\right) \end{array}$$

Parameters *ϕ*(*t*) and *υ_ϕ_*(*t*) correspond to the joint displacements and velocities of the snake-like robot. (*P_x_*(*t*), *P_y_*(*t*)) indicates the robot’s center of mass position in the global coordinate system, while *υ_t_*(*t*) and *υ_n_*(*t*) are associated with the tangential and normal velocities of the center of mass in the local frame. *θ*(*t*) represents the orientation angle, and *υ_θ_*(*t*) is the rotational velocity of the robot. Moreover, *λ_1_* and *λ_2_* are empirical constants that describe the system’s rotational dynamics.

Owing to the mechanical limitations of the snake-like robot, the model holds true solely for smaller joint distances *ϕ*(*t*). Therefore, restrictions on the joint displacements *ϕ*(*t*), velocities *υ_ϕ_*(*t*), and inputs are necessary to guarantee practical operation. These limitations are outlined as follows:

(25) $$\begin{array}{l} X=\left\{x(t)\in R^{2N_{l}+4}\left|\phi_{i}(t)\in \left[-\phi_{\max},\phi_{\max}\right]\right.,v_{\phi ,i}(t)\in \left[-v_{\phi ,\max},v_{\phi ,\max}\right],\forall i\in \prod \left[1,N_{l}-1\right]\right\}\\ U=\left.\left\{u(t)\in R^{N_{l}-1}\left|u_{i}(t)\right.\right.\in \left[-u_{\max},u_{\max}\right],\forall i\prod \left[1,N_{l}-1\right]\right\} \end{array}$$

Thus, it can be concluded that

(26) $$x(t)={\left[\begin{array}{c} \phi (t),\theta (t),p_{x}(t),p_{y}(t),v_{\phi }(t),v_{\theta }(t),v_{t}(t),v_{n}(t) \end{array}\right]}^{T}$$

And $\phi_{\max}>0$, $v_{\phi ,\max}>0$, and $u_{\max}>0$.

The lateral fluctuation gait control model is assumed to be

(27) $$\phi_{i,ref}=a\sin(\omega t+(i-1)\delta )+\phi_{0}$$

Then, the controller for the lateral fluctuating motion is

(28) $$u(t)=u_{\mathrm{ref}}(t)+k_{d}\left(v_{\phi ,\mathrm{ref}}(t)-v_{\phi }(t)\right)+k_{p}\left(\phi_{\mathrm{ref}}(t)-\phi (t)\right)$$

Among others, $v_{\phi ,\mathrm{ref},i}(t)=\frac{d}{dt}\phi_{\mathrm{ref},i}(t),\;\mu_{\mathrm{ref},i}(t)=\frac{d^{2}}{dt^{2}}\phi_{\mathrm{ref},i}(t)$.

The objective of this section is to maximize the forward speed of the snake-like robot while maintaining low energy consumption. To achieve this, a cost function is formulated that is both simple and physically intuitive. Thus, −*υ*(*t*) is introduced as the optimization objective for the Model Predictive Control (MPC) problem, seeking to minimize the cost. This forms the basis of the economical MPC-based motion control algorithm for snake-like robots proposed in this paper. Given an initial state *x*(*t*) at each time step (*t*), the economical MPC during the discrete sampling interval is defined as follows:

(29) $$\begin{array}{l} \underset{u(t)\in U^{N}}{\min}J(x(t),u(t))=-\sum_{k=0}^{N}v_{t}(k|t)\\ x(0|t)=x(t)\\ x(k+1|t)=f(x(k|t),u(k|t))\\ u(k|t)\in U\subseteq R^{N_{l}-1},\;k=0,\ldots ,N-1\\ x(k|t)\in X\subseteq R^{2N_{l}+4},\;k=0,\ldots ,N \end{array}$$

In particular, the dynamic model *f*(*x*(*k*|*t*), *u*(*k*|*t*)) and the state vector *x*(*k*|*t*) of the snake-like robot in discrete time are given by Equation (26). Here, input *u*(*k*|*t*) denotes the angular acceleration of the joints, −*v_t_*(*k*|*t*) represents the terminal cost, and *U* and *X* denote the input and state constraints, respectively. The predicted optimal input at time *t*, based on the time-series prediction, is given by

(30) $$u^{*}(t)=\left[\begin{array}{c} u^{*}(0|t),\ldots ,u^{*}(N-1|t) \end{array}\right]$$

The corresponding optimal prediction trajectory can be expressed as

(31) $$x^{*}(t)=\left[\begin{array}{c} x^{*}(0|t),\ldots ,x^{*}(N|t) \end{array}\right]$$

where u^*^(*t*) and x^*^(*t*) represent the optimal input and state trajectories, respectively. At each time step *t*, employing the first element of the optimal input sequence allows the control input to be written as

(32) $$u_{\mathrm{MPC}}(t)=u^{*}(0|t)$$

### 4.2. Experimental Validation

#### 4.2.1. Simulation Results

This section presents numerical simulations and simulation models used to validate the effectiveness of the economical Model Predictive Control (MPC) method in the practical application of snake-like robots.

The robot was modeled as a chain of nine identical joints, each with a mass *m* = 1 kg, joint length *l* = 0.14 m, and rotational inertia *I* = 0.0093 kg m^2^; the joints were connected through Hooke hinges (without considering the influence of reactive torque on the Hooke hinge, which is regarded as a rigid link and an ideal connection without elasticity in the simulation) and prismatic joints, ignoring the microscopic elastic deformation of the flexible hinges. The Mecanum wheels were treated as rigid cylinders, where *r* = 0.03 m, normal friction coefficient *c_n_* = 3, and tangential friction coefficient *c_t_* = 1, and the rolling direction was controlled through UPS parallel mechanism. The ground was simulated as a rigid plane, and the roughness variation was ignored.

The setup time was *t*_0_ = 0 s, and the initial motion states of the robot were *υ_ϕ_ =* 0 m/s, *υ_n_ =* 0 m/s, and *υ_t_ =* 0 m/s. The sampling time was *T_s_* = 0.05 s, and the prediction horizon was *Np* = 20, which corresponded to a total time of 1 s. For comparison purposes, a lateral fluctuation controller was introduced with the following parameters: amplitude (*ɑ* = 0.05 m), frequency *ω =* 120 rad/s, phase difference *δ =* 40°, and joint spacing *D_0_ =* 0 m.

The cost function for designing the economical MPC is given by the energy expended during the considered movement. The average energy consumption is computed based on Equation (33):

(33) $$F(x(t),u(t))=\sum_{k=0}^{N_{p}}-v_{t}(k|t)+\varepsilon u^{T}(k|t)u(k|t)$$

where $\varepsilon u^{T}(k|t)u(k|t)$ denotes the energy expended during the considered movement.

The average energy consumption is based on the following equation:

(34) $$\bar{E}=\sum_{t=100}^{200}\frac{u^{T}(t)u(t)}{100}$$

The average asymptotic velocity is

(35) $$\bar{v}=\sum_{t=100}^{200}\frac{v_{t}(t)}{100}$$

**Figure 16:**
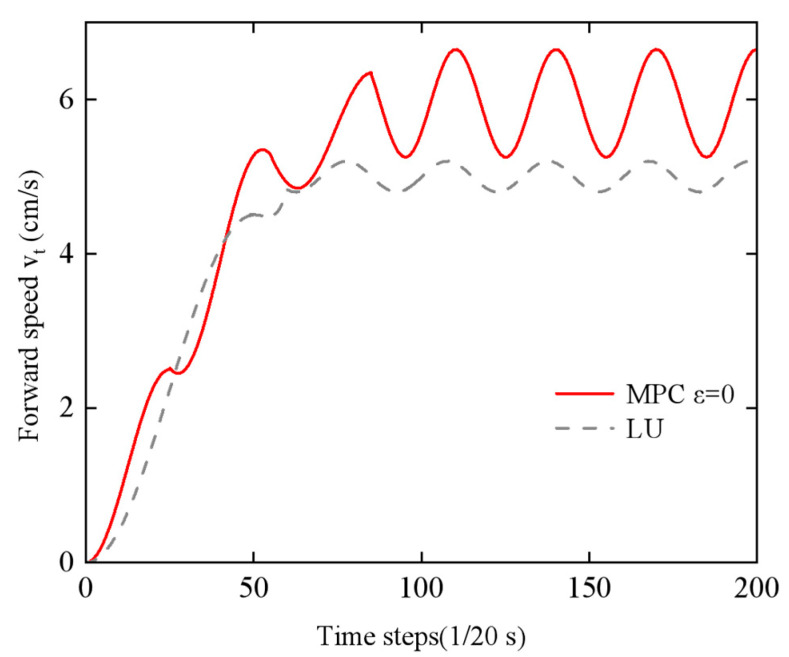
Comparison of forward speed of economy MPC with lateral swing controller for *ε* = 0.

**Figure 17:**
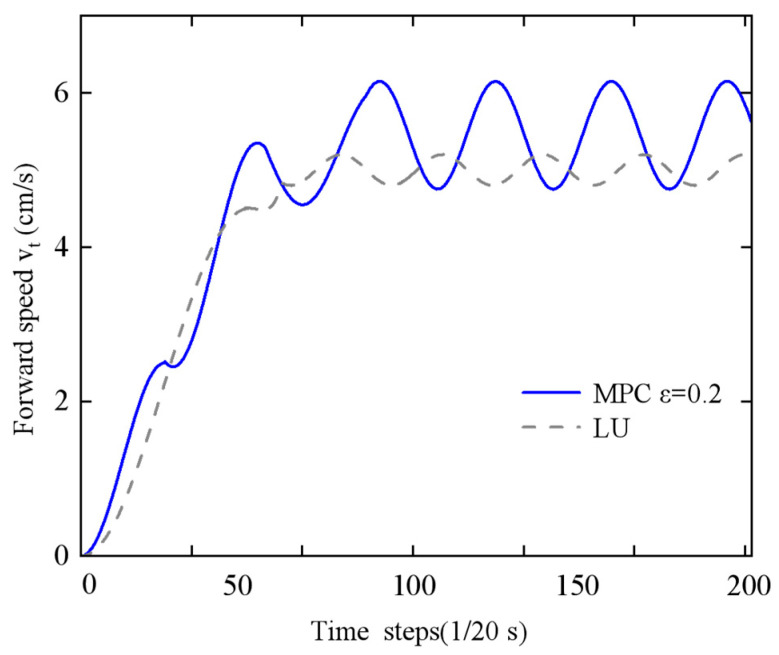
Comparison of forward speed of the economical MPC with lateral fluctuation controller for *ε* = 0.2.

In this study, the performance differences between two control strategies—Economic Model Predictive Control (EMPC) and lateral undulation (LU)—were compared and analyzed for the motion control of the snake-like robot. To provide a comprehensive evaluation of the control performance, the experiments were carried out in two dimensions: motion speed and energy consumption. Figure 16 presents a comparison of the periodic motion speeds achieved by the two controllers (economic MPC with *ε* = 0 and lateral undulation controller) after a 100-step transition phase, while Figure 17 shows the comparison of periodic motion speeds when the remaining conditions are the same but *ε* = 0.2 (economic MPC with *ε* = 0.2 and lateral undulation controller).

The selection of *ε* = 0.2 reflects a multi-objective optimization strategy, analogous to the Pareto frontier-based framework proposed by Zhou et al. [35], who addressed energy–efficiency trade-offs in hybrid powertrain design. By systematically varying *ε*, we constructed a solution set where improvements in speed and energy consumption are non-dominated, with *ε* = 0.2 representing a practical compromise that aligns with biological locomotion efficiency.

The experimental results (Figure 16) indicate that the snake-like robot under EMPC demonstrates a significant speed advantage, with an asymptotic average speed increase of 28% compared to the LU controller. Further analysis reveals that, traditionally, speed enhancement is often accompanied by an increase in energy consumption. However, this study achieves a breakthrough by incorporating an energy optimization strategy. As shown in Table 2, while the standard EMPC scheme increases energy consumption by 22% during periodic steady motion, the improved EMPC scheme not only further increases the asymptotic mean speed by 18% but also reduces energy consumption by 7% when combined with parameter optimization adjustments.

To further quantify the energy–speed trade-off, the Cost of Transport (CoT), defined as the ratio of energy consumption to locomotion speed (J·s/m), is introduced. As shown in Table 2, the optimized EMPC scheme (*ε* = 0.2) achieves a 17.4% reduction in the CoT compared to the standard EMPC (*ε* = 0) (3.47 J·s/m vs. 4.20 J·s/m), demonstrating improved energy efficiency.

Specific quantitative indicators reveal that the average energy consumption, calculated using Equation (33) during a stable motion cycle of 100 time steps, shows that the optimized EMPC scheme improves the ratio of the CoT by 17.4%. This improvement is attributed to the adaptive capabilities of the EMPC framework, which can dynamically adjust the motion gait parameters based on real-time environmental conditions. Furthermore, energy consumption is substantially decreased through the improved design of the energy term, while preserving high mobility efficiency.

This study demonstrates that the economical MPC method overcomes the traditional linear trade-off between speed and energy consumption by introducing an energy optimization mechanism. It provides a novel control paradigm for snake-like robots, balancing both motion efficiency and energy economy.

#### 4.2.2. Prototype Testing

**Figure 18:**
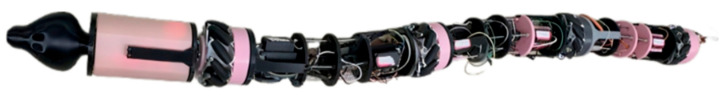
Snake-like robot prototype.

To verify the overall performance of the Economic Model Predictive Control (EMPC) algorithm in practical settings, this study conducted lateral undulation motion control experiments using a physical prototype of a snake-like robot (Figure 18). The experimental design adhered to a dual validation principle: first, ensuring that all control parameters matched those used in the simulation environment, thereby providing a basis for comparable algorithm verification; and second, systematically evaluating the actual effectiveness of the control strategy in optimizing the trade-off between energy consumption and locomotion speed through a parameter space exploration experiment.

**Figure 19:**
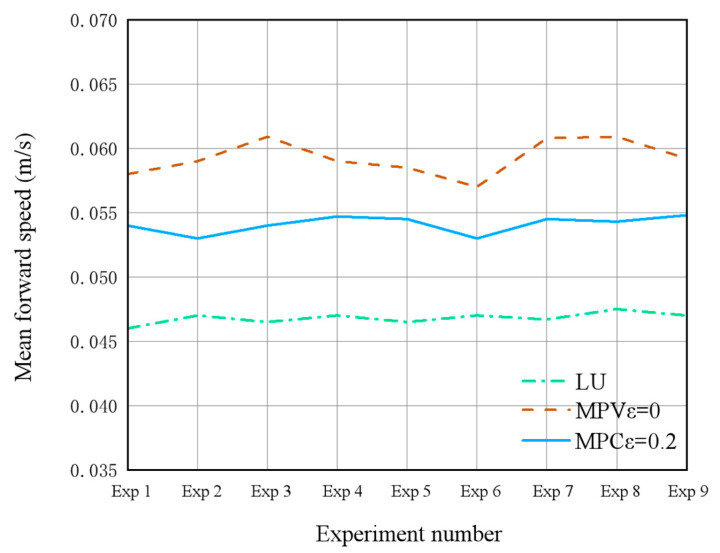
Average forward speed of the center of mass of the snake-like robot prototype under different schemes in three sets of experiments.

Three control schemes were tested: the standard EMPC, the optimized EMPC, and a lateral undulation (LU) controller. Each scheme underwent nine repeated trials to mitigate the impact of random errors. In the optimized EMPC scheme, dynamic energy management was achieved through the online adjustment of the objective function weights. Figure 19 presents the comparative data of the robot’s center-of-mass forward velocity under the three control strategies, while Table 3 quantifies the corresponding energy consumption metrics.

The experimental results indicate that the standard EMPC scheme increases the average speed by 27.2% compared to the LU controller (0.0598 m/s vs. 0.0470 m/s). The optimized EMPC scheme maintains a speed of 0.0531 m/s, representing an 11.2% reduction from the standard EMPC but a 12.9% increase over the LU controller, while reducing energy consumption by 7.3% (0.2147 J vs. 0.2317 J). Notably, the Cost of Transport (CoT) improved by 18% under the optimized EMPC (4.04 J·s/m vs. 4.93 J·s/m), demonstrating the dynamic parameter adjustment mechanism’s effectiveness.

Physical testing highlighted non-negligible deviations from simulation predictions, primarily attributed to thermal effects on motor efficiency and terrain-induced friction variations. While the absolute performance metrics were affected, the adaptive control framework maintained functional viability under real-world conditions. These insights provide a clear direction for the future optimization of the electromechanical system. This strategy enables up to an 18% extension in operational endurance without compromising the minimum speed requirement, which holds substantial engineering significance for energy-constrained field-deployable robotic systems.

## 5. Conclusions

This study presents a comprehensive approach to enhancing the performance of snake-like robots in complex exploration and rescue missions, with three key contributions:

(1) Bio-inspired Compliant Mechanism: A novel hybrid joint design combining compliant mechanisms and Mecanum wheels was developed. Supported by screw theory-based kinematics and Newton–Euler dynamics modeling, this design significantly improves terrain adaptability and mobility.

(2) Smooth Multimodal Gait Control: A dual-chain central pattern generator (CPG) network, integrated with linear smoothing algorithms, was proposed to enable seamless gait transitions. This approach addresses the signal discontinuity issues commonly encountered in conventional methods.

(3) Energy–Speed Co-optimization: An economic Model Predictive Control (MPC) framework was established to overcome the limitations of traditional single-objective optimization strategies. In simulations, this framework achieved a 7% reduction in energy consumption (0.1952 J vs. 0.2107 J) and an 18% increase in average forward speed (0.0563 m/s vs. 0.0478 m/s) compared to traditional controllers. Prototype experiments demonstrated a 12.9% speed improvement and 7.3% energy reduction, validating the approach’s practical efficacy.

Collectively, these innovations—spanning bio-inspired compliant joints, adaptive gait control, and energy–speed co-optimization—significantly advance the operational efficacy and deployment readiness of snake-like robots in unstructured environments. However, the precise energy-saving mechanisms underlying this synergy remain partially unresolved. Specifically, the distinct roles of mechanical compliance (e.g., anisotropic friction via Mecanum wheels) versus control intelligence (e.g., MPC-driven trajectory optimization) in enhancing energy conversion efficiency require deeper mechanistic validation. While the Cost of Transport (CoT) metric (Table 2 and Table 3) indirectly supports improved efficiency, direct quantification through power flow analysis—such as correlating motor input power with net forward propulsion—is absent.

To bridge these gaps, future work will prioritize the following:

(1) Decoupling mechanical and control contributions via ablation studies (e.g., rigid-joint variants) to isolate MPC-driven energy savings;

(2) Developing a dynamic power model that quantifies energy transfer pathways by integrating bionic mechanics (e.g., spinal elasticity) with adaptive control (e.g., phase-smoothing);

(3) Validating synergistic principles through multiphysics co-simulations (e.g., ADAMS-CoppeliaSim) to uncover governing laws of structure–control co-optimization.

These initiatives will establish a foundational theory for energy-aware bio-inspired robotics, enabling the robust design of high-efficiency systems in complex terrains.

## Figures and Tables

**Table 1:** CPG parameters for generating serpentine gait movements.

Horizontal Chain (Steering)		Vertical Chain (Pitching)	
Notation	Parameter Value	Notation	Parameter Value
$a_{c}$	0	$b_{c}$	0
$\phi_{h,ij}$	$\frac{1}{3}\pi$	$\psi_{h,yx}$	$\frac{1}{2}\pi$
$a_{H}$	1	$\omega_{h}$	$\frac{1}{5}\pi$
$r_{x}$	1		

**Table 2:** Simulation results: comparison of energy consumption, speed, and Cost of Transport (CoT) for MPC and lateral undulation controllers.

Control Methods	Asymptotic Average Speed (m/s)	Specific Value	Average Energy Consumption (J)	Specific Value	CoT (J·s/m)	Specific Value
*MPC*|*ε* = 0	0.0612	128%	0.2573	122%	4.20	95.24%
*MPC*|*ε* = 0.2	0.0563	118%	0.1952	92.64%	3.47	78.68%
Transverse Fluctuation Controller	0.0478	100%	0.2107	100%	4.41	100%

**Table 3:** Experimental results: comparison of energy consumption, speed, and Cost of Transport (CoT) for MPC and lateral undulation controllers.

The Average Forward Speed for the Three Sets of Experiments				The Average Energy Expenditure of the Three Sets of Experiments		
**Control Methods**	Average Forward Speed (m/s)	Specific Value	Average Energy Consumption (J)	Specific Value	CoT (J·s/m)	Specific Value
*MPC*|*ε* = 0	0.0598	125.4%	0.2830	122%	4.73	95.94%
*MPC*|*ε* = 0.2	0.0531	112.9%	0.2147	92.7%	4.04	81.95%
Transverse Fluctuation Controller	0.0470	100%	0.2317	100%	4.93	100%

## Data Availability

Data will be made available on request.
